# Analysing variation in *Drosophila* aging across independent experimental studies: a meta-analysis of survival data

**DOI:** 10.1111/acel.12123

**Published:** 2013-07-22

**Authors:** Matthias Ziehm, Matthew D Piper, Janet M Thornton

**Affiliations:** 1 EMBL – European Bioinformatics InstituteWellcome Trust Genome Campus, Hinxton, Cambridge, CB10 1SD, UK; 2 Institute of Healthy Ageing, University College LondonGower Street, London, WC1E 6BT, UK

**Keywords:** aging, bioinformatics, demography, *Drosophila*, meta-analysis, survival data

## Abstract

Survival records of longevity experiments are a key component in research on aging. However, surprisingly there have been very few cross-study analyses, besides comparisons of median lifespans or similar summary information. Here, we use a large set of full survival data from various studies to address questions in aging, which are beyond the scope of individual studies. We characterize survival differences between female and male flies of different genetic *Drosophila* strains, showing significant differences between strains. We further analyse the variation in survival of control cohorts recorded under highly similar conditions within different *Drosophila* strains. We found that overall transgenic constructs of the UAS/GAL4 expression system which should have no effect (e.g. a GAL4 construct alone) extend lifespan significantly in the w1118 strain. Using a large data set comprised of various studies, we found no evidence for larger lifespan extensions being associated with shorter lifespans of the control in *Drosophila*. This demonstrates that lifespan extending treatments are not purely rescuing weak backgrounds.

## Introduction

Research on the biology of aging has been conducted for more than a century, and the age of death, as an unambiguously defined, universally occurring event, is the most commonly used phenotype. While research on aging is a long-established field, there have been very few studies combining survival data from multiple studies. Use beyond the primary study is generally limited to comparing changes in mean lifespan or similar high-level summary statistics. This is not least due to the fact that survival data from lifespan experiments have not been publicly accessible. A second factor complicating meta-analyses is that survival is influenced by many environmental factors and these are not standardized across different laboratories. Thus, meta-analyses are limited to either large enough subsets of data with identical conditions or involve the application of methods accounting for varying additional factors. Here, we demonstrate the power of meta-analysis of survival data to address questions in research on aging that are beyond the scope of individual experiments using data from the newly established database SurvCurv (Ziehm & Thornton, this issue).

We have focussed our analyses on *Drosophila melanogaster*, a powerful tool for discovery and widely used model organism in research on aging. Research in *Drosophila* is carried out in various different genetic strains. Here, we concentrate on two commonly used strains called w1118 and wDah. The *white*^1118^ (w1118) strain originates from the wild caught Oregon R strain, but contains a spontaneous partial deletion in the *white* (*w*) gene discovered by R. Levis (Bingham, 1980; Hazelrigg *et al*., 1984), which leads to white instead of the normal red eyes. *white*^Dahomey^ (wDah) is a strain created in the Partridge Lab and was derived by repeated backcrossing of the mutated *w* gene from w1118 into the outbred wild-type Dahomey strain. The Dahomey strain was originally collected in Dahomey, now Benin (Puijk & de Jong, 1972), and has been maintained since then in large population cages with overlapping generations.

In *Drosophila*, as in many other species, different sexes have different average lifespans, with the females in general being the longer lived gender. We asked: Are gender differences in *Drosophila* aging strain-specific and how do they compare? When are the differences in survival or mortality between the sexes biggest? We also examined the variation in lifespan of female control cohorts of highly similar conditions in different strains and asked how large is the variation and can we identify hidden factors explaining some of the variation? And how do different genetic strains compare to each other? For addressing these questions, we only used data in which factors not investigated, such as temperature or food conditions, did not vary largely. Thus, the intrinsic variations become more tractable. While this is the best practically possible approach, one has to keep in mind that even very small differences in assay conditions can cause large effects. If the lifespan of control cohorts varies, is this problematic? Based on our large data set of survival records, we tested whether lifespan extension correlates with lifespan of the control.

## Results

### Female–male survival differences

Female life expectancy in humans is higher than that of men in almost all regions around the world. Similarly, females are the longer lived gender in *Drosophila* and many other species with some notable exceptions. In most birds, for example, males live longer than females, and in *Caenorhabditis elegans*, males outlive hermaphrodites. The question of why different sexes have different lifespans is age-old, and numerous studies have identified genes affecting aging in a sex-dependent manner. Various reviews have been published focussing on sex differences in longevity with respect to certain biological systems such as telomere biology (Barrett & Richardson, 2011) or apoptosis (Tower, 2006), or discussing interventions with sex-specific effects (Burger & Promislow, 2004). However, which of the known differences between the sexes are causing the observed differences in longevity is still unclear and a topic of ongoing debate today. Therefore, we wanted to characterize the differences between female and male survival in *Drosophila* and identify the time points of maximal difference in survival and mortality. These time points are crucial to optimally contrast general gender differences with gender differences relevant to aging using molecular analyses.

**Figure 1:**
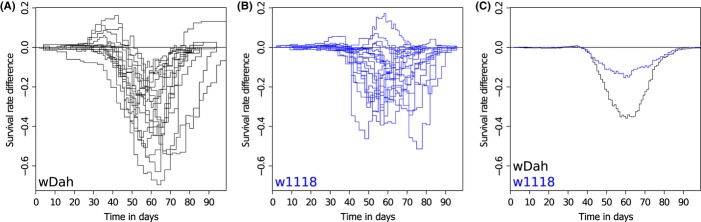
Gender difference profiles. Individual female–male difference plots of wDah (A) and w1118 (B), showing the variation between experiments. (C) Female–male difference profiles of wDah (black) and w1118 (blue), that is, difference plot of the respectively combined female and male cohorts. Positive values indicate a male survival advantage, whereas a line below zero indicates a survival disadvantage for males, that is, a survival advantage for females. Plots are based on a total of 2507 female and 2216 male wDah flies as well as 3598 females and 3486 males of w1118.

Using the collected data sets, we explored gender differences in two *D. melanogaster* strains, w1118 and wDah. A Cox proportional hazards (CoxPH) analysis (Cox, 1972) of the gender factor in the two strains indicated a 1.39-fold increased mortality rate for males in w1118 [*P*-value < 10^−15^; 95% confidence interval: (1.32,1.46)] and a 2.34-fold increased mortality rate for males in wDah [*P*-value < 10^−15^; 95% confidence interval: (2.19,2.50)], indicating a stronger gender difference in wDah. Taken together, in our data set, we found males to be consistently shorter lived than females and gender difference in survival to be larger in the wDah background compared with w1118. Figure 1A,B shows individual difference plots between paired cohorts of female and male survival studies, illustrating the variation in female–male survival differences across cohorts. Difference plots are a derived representation, which instead of showing absolute survival or mortality curves shows only the differences between pairs of them, in this case male and female. Here, the curve shows the male deviations from the female, that is, a line below zero indicates a male survival disadvantage. We then built strain- and gender-specific metacohorts of the available data. Using these metacohorts, we created female–male survival difference plots for each strain (Fig. 1C, see Fig. S1 for the corresponding mortality profile and Table S1 for a list of the underlying SurvCurv IDs). These profiles can be seen as fingerprints of the gender difference with respect to longevity, characterizing the difference phenotypically in a time-resolved manner. While many interventions affect lifespan in a sex-dependent way (Burger & Promislow, 2004), in our dataset female–male lifespan profiles are consistent between wild-type controls and experimental mutant lines within each strain, that is, genetic background (Fig. S2). The maximal difference in survival rate between the genders in both strains was determined to be around day 60. An alternative view on survival data is to calculate mortality rates, also called hazard rates. These show the instantaneous risk of dying at a certain point in time in contrast to the cumulative probability of surviving up to a certain age shown in the survival plots. The maximal difference in mortality (Fig. S1) between the genders is earlier than the maximal difference in survival, for example at around day 44 for wDah. While the origins of these differences remain unknown and the analysis of the combined data agrees with the previous notion that females are longer lived than males in *Drosophila*, we generalized these observations, quantified effect sizes and determined time points of maximal differences. This knowledge enables us to select optimal time points for sampling gender differences related to aging for molecular analysis, for example one might want to sample at an early time point with no significant difference in survival or mortality between the genders and one with large differences to discriminate between general gender differences and those relevant to aging.

### Variation of controls

Using SurvCurv, we have analysed the variation in the control conditions for different *D. melanogaster* strains. We have restricted the analysis to female control cohorts at 25 °C and to the most common feeding paradigm in our data set, 1SY, an agar-based food mix containing sugar and 100 g L^−1^ yeast. Median lifespans for the strains wDah and w1118 varied between 32 and 75 and 46 and 80, respectively. These within-strain variations of the lifespan under defined control conditions render the direct comparisons of absolute survival of treatment conditions imprecise and vulnerable to misinterpretation.

**Figure 2:**
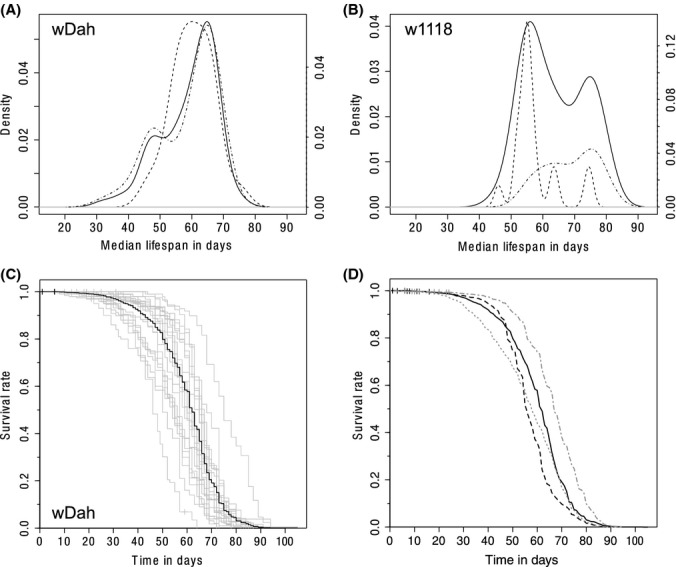
Variation of controls and average control survival curves in *Drosophila*. Density distribution of median lifespans in 1SY fed female controls (25 °C) of the *white* Dahomey (wDah) strain (76 cohorts) (A) and w1118 (45 cohorts) (B). The density distribution of w1118 suggests that the w1118 consist of two distinct groups, which could be identified as controls not containing any genetic alterations (dashed, left group) and those which contain genetic constructs (dash-dotted, right group), which should be inactive. These are UAS construct alone, GAL4 construct alone or a noninduced GeneSwitch construct. (C) Survival curves of 22 female wDah control cohorts without constructs on 1SY food 25 °C and the survival curve estimated from the pooled data (black). (D) Survival Curves estimates from pooled 25 °C 1SY fed female control cohorts for various *Drosophila melanogaster* strains: wDah no constructs (solid, 22 cohorts), w1118 no constructs (dashed, 19 cohorts), wDah with constructs (dotted grey, 54 cohorts) and w1118 with constructs (dash-dotted grey, 26 cohorts).

We next examined the density distribution of the individual medians of the control cohorts and found them to be non-normally distributed [Shapiro–Wilk test *P*-values < 0.005 (Shapiro & Wilk, 1965)] in both strains, with the strongest bimodal distribution present in w1118 (Fig. 2A,B solid lines). Thus, we examined whether any of the existing annotations would result in separate more unimodal distributions. In particular, we assessed the effects of theoretically inactive genetic constructs such as UAS or GAL4 constructs alone or noninduced GeneSwitch–GAL4 constructs in w1118 controls using a CoxPH model with an ‘inactive’ construct factor. The UAS/GAL4 transcriptional system (Brand & Perrimon, 1993) has two transgenic elements: (i) a promoter from *Drosophila* cloned upstream and driving expression of the GAL4 transcriptional activator from *Saccharomyces cerevisiae* and; (ii) a promoter containing GAL4 binding sites (upstream activator sequence; UAS) upstream of a transgene of interest. Separately, the two transgenes should have no effect on *Drosophila* as GAL4 is a yeast transcription factor and the UAS construct alone should not be transcribed in the absence of GAL4. Alternatively, a variant of the GAL4 transcription activator, GeneSwitch–GAL4, is used to allow time-controlled expression of genetic constructs. This variant is inactive until the drug RU486, also known as mifepristone (CHEBI:50692), is fed.

We found the ‘inactive’ construct factor to be associated with a strongly reduced mortality rate (CoxPH coefficient −0.8999, corresponding to a relative mortality risk of 0.407, i.e. a 2.5-fold reduced mortality rate, *P*-value < 10^−15^). The precise estimate of the change in mortality rate might be less certain in this case due to the fact that the proportional hazards assumption underlying the CoxPH model is rejected (*P*-value < 10^−15^) and a modest deviation from the assumption (*r *= 0.17) is detected. However, even taking into account a larger uncertainty due to the violated model assumptions, a clear reduction is reliably indicated, because of the strength of the effect. Moreover, splitting the w1118 controls into two groups by the presence or absence of these theoretically inactive genetic constructs resulted in two separate distributions, one peaking under each of the peaks of the combined distribution (Fig. 2B dashed lines). Interestingly, the controls containing ‘inactive’ genetic constructs characterize the longer lived group in w1118. In wDah, the distributions of these two groups have a large overlap, and the peak of the distribution of controls with constructs is at a higher age compared with the peak of the distribution of control without constructs (Fig. 2A dashed lines). This distribution of controls with constructs is, however, bimodal itself. It has a secondary peak earlier in time, which results in an overall higher mortality rate of the controls with ‘inactive’ constructs in wDah as evident from a CoxPH analysis (CoxPH coefficient 0.1624, corresponding to a 1.18-fold slightly increased mortality rate, *P*-value < 10^−12^). We did examine further annotated variables and only found the date of the experiment to be a significant covariate (*P*-value < 10^−11^), albeit with marginal influence (CoxPH coefficients < 10^−3^, corresponding to a relative risk of almost exactly 1). CoxPH estimates for w1118 and wDah including a date covariate are −0.949 and 0.163, respectively, corresponding to relative mortality risks of 0.39 and 1.18, respectively. We also examined a partitioning of UAS constructs versus GAL4 constructs, but a relatively high number of cases with inactive GeneSwitch–GAL4/UAS rendered this analysis problematic as no partitioning is possible, and decreasing numbers per condition are limiting the analyses. Possible causes for the remaining variations include differences between food and food ingredient batches, differences in handling between experimenters or temporal differences in noncontrolled environmental factors, such as noise or atmosphere including air pressure and oxygen concentration.

While we could not identify markers for all potential different subgroups, that is, markers explaining all the variation, we show that the insertion of ostensibly inactive genetic constructs often leads to significant changes in median lifespan. This might result from so-called leakiness of the construct, for example a UAS transgene construct, which is lowly expressed even in the absence of GAL4, or from positional effects of the insertion, which might interrupt a gene or change the expression of a certain genomic region. Our results clearly demonstrate that a full set of controls needs to be incorporated into each aging study, because insertion site effects or construct–genotype interactions on their own can give rise to lifespan extension.

Survival curves of all the individual control cohorts without any genetic constructs for wDah are shown in Fig. 2C illustrating the variation (Fig. S3 for wDah with ostensibly inactive genetic constructs and w1118 with and without constructs). The black curve is calculated by pooling the survival data from the individual, greyed out, survival curves, thus representing an average survival behaviour. We calculated pooled survival curves for 1SY fed female controls of both strains with and without constructs (Fig. 2D). These curves, representing an average survival behaviour for the defined condition, allow us to compare the strains with higher confidence survival curves, due to the vastly increased number of observations compared with single studies, rendering the estimates more robust to random influences. These curves indicate that wDah and w1118 follow different survival patterns, and the one of w1118 is characterized by a longer plateau and a more rapid mortality. This indicates less within-strain variation, which could be due to the fact that w1118 is the more highly inbred line, with largely reduced genetic variation between individuals. Pooled survival data of controls, like the one presented here, can also be used as so-called historical controls (see also Ziehm & Thornton, this issue). Note: the historical controls available in SurvCurv are defined like the one presented here but include only public data).

### Lifespan extensions do not depend on the short-lived controls

The primary interest of research in aging is to understand interventions that slow normal aging. However, the rescue of a sick short-lived control condition to a normal-lived treatment condition also presents a relative lifespan extension. While every effort should be made to avoid this when conducting research on aging, it might not be obvious in all cases whether a cohort of animals is healthy normal and still short living, or sick or abnormal and thus short living. Linnen *et al*. (2001) elegantly demonstrated that at least some standard laboratory strains might have to be considered ‘sick’ compared with wild animals, due to the implicit selection regime applied during stock maintenance, driving them towards early reproduction and short lifespan.

An interesting question is thus whether larger lifespan extensions in *Drosophila* are generally associated with shorter lived control cohorts. If this were the case, differences in absolute survival of the controls would be especially problematic. A study by Orr & Sohal (2003) is sometimes cited as evidence in favour of this association. They examined lifespan extensions and lifespan of control of nine different studies on superoxide dismutase (SOD) or closely related proteins and found ‘a clear negative correlation’ between percentage lifespan extension and lifespan of the control. However, Spencer *et al*. (2003) showed around the same time that SOD over-expression extended lifespan in six of 10 long-lived backgrounds in females, four of which are large extensions. This contradicts Orr's correlation.

Here, we examine whether there is a correlation between the magnitude of lifespan change and lifespan of the control across all experimental pairs in *Drosophila* in the SurvCurv database. However, instead of using the percentage change, we use the difference in median lifespan, which is a better measure to use. As the percentage change is defined by lifespan extension divided by lifespan of the control, a constant absolute lifespan extension at various control lifespans would inevitably result in a negative correlation of percentage change and control lifespan.

Using 499 pairs of control and treatment from the database, we did not find any correlation (Spearman's correlation coefficient *r*_S_* *= 0.03 after excluding outliers [*r*_S_* *= −0.054 including outliers]). Outliers were determined by using the median absolute deviation (MAD) estimator, using mean ± 3.3 times the MAD estimator as boundaries. For a normal distribution, this would include approximately the central 99.9% of the data. Assuming normally distributed values, a power analysis shows that with our dataset of 499 data points, we would detected correlation coefficients as low as 0.22 with a significance level 0.01 and a power of 0.99, that is, in 99% of all cases. This shows it is very unlikely that we are missing any larger correlation just by chance. We conducted a number of additional tests, described below, to ensure some potential factors are not causing the observed results.

**Figure 3:**
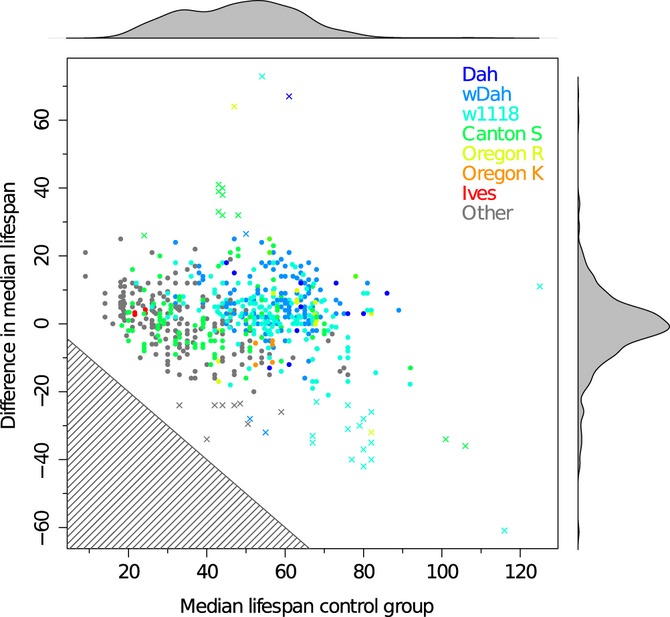
Correlation analysis of median lifespan and lifespan extension in *Drosophila*. Correlation of median control lifespans with difference in median lifespan. *x* indicate outliers excluded for the correlation. Outliers were defined as being more than 3.3 times the median absolute deviation (MAD) estimator away from the mean. Different strains are indicated by different colours, and density distribution of the data is shown along the axis. The shaded area in the main diagram indicates the range impossible combinations, because animals would be nonviable. Lifespans are given in days.

We analysed the correlation excluding in addition to the outliers all control–treatment pairs that do not show a significant change in lifespan to ensure that these do not pull the correlation to zero. These analyses also showed no significant correlations (Spearman's correlation coefficient *r*_S_* *= −0.08 after excluding outliers and all pairs with log-rank *P*-values ≥ 0.01 and *r*_S_* *= −0.09 after excluding outliers and all pairs with Wilcoxon rank-sum test *P*-values ≥ 0.01). For the two most populated strains, wDah and w1118, Spearman's correlation coefficients of −0.151 and −0.088 were determined, respectively, indicating that there is also no significant strain-specific correlation (*P*-values > 0.01). To further test this important finding, we included median lifespan values of an additional 247 control–treatment pairs from 18 articles not contained in the database. Median lifespan values were extracted from the literature; mean values were used where only these were given. Using this extended data set confirmed that there is no tendency for larger lifespan extensions based on shorter lived controls [overall *r*_S_* *= 0.03 (*r*_S_* *= −0.051 including outliers), see Fig. 3, Fig. S4 for equivalent analysis on percentage change]. While the absence of correlations could be confirmed in wDah (*r*_S_* *= 0.04) and no correlation was found in the Canton S strain either (*r*_S_* *= 0.024), a modest, significant correlation was found in the w1118 strain (*r*_S_* *= −0.36, *P*-value < 0.001). These strain-specific results originate from less data than the overall correlations (1/5 to 1/4 of the overall data), thus offering less confidence. Still they indicate that the lack of overall correlation is not the results of opposing correlation trends in different strains.

Because lifespan shortenings, that is, pairs with negative differences, might mask a correlation among the other pairs, we analysed the correlation of difference in median lifespan and median lifespan of the control excluding all pairs with negative differences. This left 314 control–treatment pairs from the database and 172 from literature, for which we found no correlation either [Spearman's correlation coefficient *r*_S_* *= −0.04 after excluding outliers (including outliers did not change the correlation coefficient)].

Finally, we performed pairwise CoxPH analyses of all 499 control–treatment pairs from the database to determine the respective proportional hazards coefficient, whose exponential indicates the relative mortality rate. We then analysed the correlation of median lifespan of the control and the CoxPH coefficient and also found no correlation [Spearman's correlation coefficient *r*_S_* *= −0.067 after excluding outliers (*r*_S_* *= −0.005 including outliers), see Fig. S5]. To further ensure that the lack of overall correlation is not due to opposing trends in different strains, we again also determined strain-specific correlation coefficients for the two most populated strains, wDah and w1118. We found no significant correlation [Spearman's correlation coefficients of *r*_S_* *= 0.11 (*P*-value > 0.1) and r_S_* *= −0.095 (*P*-value > 0.1) respectively] confirming there are no opposing correlation trends in the different strains. Overall these results clearly demonstrate the absence of any correlation between lifespan extension and lifespan of the control.

## Discussion

To date, there have been hardly any independent analyses of survival data from separate studies, as well as widespread concerns that these data cannot be combined. Here we have shown that with care and annotated accessible survival data, such as provided by SurvCurv, meta-analyses are possible and can reveal new insights. Generic factors, for example sex, strain, feeding regime, can be examined by combining data from different experiments, which increases the number of observations and thus gives higher confidence results. Moving forward the ability to combine observations on various interventions from separate experiments on a signalling, metabolic or regulatory pathway might be used to examine their interactions and maybe generate hypotheses on the impact of combinations of mutants. While increasing amounts of available data will render more and more constraint conditions common enough to allow meta-analyses, standardized operating procedures, eliminating or at least reducing the variability in nonexperimental factors, would be helpful to allow easier combination of experiments and refined results.

Here, we used numerical survival data from *Drosophila*, the SurvCurv web interface as well as specific analysis scripts to address questions, which were otherwise very difficult or impossible to approach. We showed that it is possible to create phenotypic gender difference profiles of different genetic backgrounds, which can guide the selection of an experimental system and time points of sampling. These findings are currently based on a limited number of studies, and a larger data basis will enable further refinement of these profiles. A more comprehensive comparison of strains, which will be possible with increasing amounts of data, will be interesting future work, especially if coupled with large scale molecular characterization data sets, like genome or transcriptome sequencing or proteome data. A combination of such data could allow for association studies in model organisms. Furthermore, we quantitatively assessed the variations between female controls of highly similar conditions and showed a highly significant effect on lifespan of the majority of UAS and GAL4 constructs in w1118. This clearly demonstrates that a full set of genetic controls needs to be incorporated into each lifespan study on aging.

The large set of survival records enabled the definition of average survival curves based on the joint set of observations, which we used to compare different *Drosophila* strains. Like in toxicity and cancerogenicity studies, pooled controls can also be used as additional, so-called historical controls to put individually measured lifespans into a bigger picture by a three-way comparison between historical control, measured control and measured treatment condition.

The collection of survival records of treatments and controls enabled us also to examine the relationship between median lifespan of the control and lifespan extension to clarify this important relationship, which had previously only been addressed on a small set of SOD related studies by Orr & Sohal (2003). They found ‘a clear negative correlation’; however, Spencer *et al*. (2003) found large lifespan extensions in SOD over-expressions in long-lived backgrounds, thus leading to an unclear situation. We found no evidence for larger lifespan extensions to occur more often with shorter lived controls, which was further confirmed by including literature-mined median and mean lifespan values, analysing correlation of only significantly different changes, only positive changes, as well as analysing the correlation of median lifespan of the control and CoxPH coefficients all showing no correlation. This clearly demonstrates that there is no evidence that lifespan extensions through treatments in *Drosophila* would in general reflect only rescues of shortened lifespan. The question whether it is true in other organisms, such as the nematode *C. elegans* or mouse, however, requires separate examination and certainly is an interesting one to address.

## Experimental procedures

### Data

All analyses were based on data from the SurvCurv database (Ziehm & Thornton, this issue). Nonpublicly accessible data, for example copyrighted or unpublished, were included in any analyses where anonymity of these data could be assured. Additionally, median and mean lifespan values from 18 articles listed in Data S1 (Supporting information) were used in the correlation analysis of lifespan extension and lifespan of the control.

### Statistical analyses and visualizations

Statistical analyses, tests and visualizations were performed using R (R Development Core Team, 2010) and the R survival package (Therneau, 2009). Modified R scripts of SurvCurv were used for all survival curves, mortality curves, difference plots and CoxPH analyses. The proportional hazards assumption was tested for all applications of the CoxPH model using cox.zph of the survival package (Grambsch & Therneau, 1994). Deviations from the assumed independence of transformed survival time and the scaled Schoenfeld residuals were determined. While the proportional hazards assumption was significantly rejected in many cases, this is in part due to the large data sets used here, which allow for significant detection of very small deviations. Therefore, it is very important to check the extent of the deviations from the assumed *r *= 0. We found the deviations were mostly small (|*r*| < 0.1), with exceptions noted. Thus, the estimates of the CoxPH model can be assumed to be reliable within the normal margin of error. Density distributions are estimated using a Gaussian kernel with a smoothing bandwidth computed by Silverman's rule of thumb [Silverman, 1986, p. 48, equation (3.31)]. Correlations are Spearman's correlations.

## Supporting Information

**Fig. S1** Female-male mortality difference profiles of wDah (black) and w1118 (blue). **Fig. S2** Gender Difference Profiles of Control and Mutants separately for wDah (A) w1118 (B). **Fig. S3** Variation of female Drosophila controls in different strains. **Fig. S4** Correlation analysis of median lifespan and percentage lifespan extension in flies. **Fig. S5** Correlation analysis of median lifespan and Cox proportional hazards coefficient for control-treatment difference in flies. **Table S1** SurvCurv IDs used for the gender difference plots (not all are publicly available).

Data S1 References of articles from which median or mean values were extracted for the analysis lifespan extension and lifespan of the control.
