# Supplementary material for: Analysing variation in Drosophila aging across independent experimental studies: a meta-analysis of survival data
Source: Aging Cell. 2013 Jul 22;12(5):917–22. doi: 10.1111/acel.12123 (PMC3963443; doi:10.1111/acel.12123)
Supplement: Supplementary file 1 — Fig. S1 Female-male mortality difference profiles of wDah (black) and w1118 (blue). Fig. S2 Gender Difference Profiles of Control and Mutants separately for wDah (A) w1118 (B). Fig. S3 Variation of female Drosophila controls in different strains. Fig. S4 Correlation analysis of median lifespan and percentage lifespan extension in flies. Fig. S5 Correlation analysis of median lifespan and Cox proportional hazards coefficient for control-treatment difference in flies. Table S1 SurvCurv IDs used for the gender difference plots (not all are publicly available). [file acel0012-0917-sd1.pdf]

Supporting Information

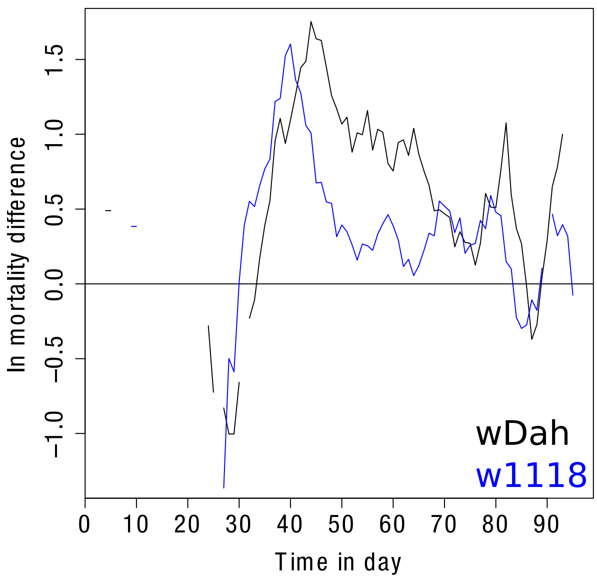

Figure S1: **Female-male mortality difference profiles of wDah (black) and w1118 (blue)**, i.e. mortality difference plot of the respectively combined female and male cohorts (a +-2 sliding window smoothing has been applied). If the line is above zero this indicates a higher male mortality, where as if it below it indicates a lower male mortality, i.e. a higher female mortality. Plots are based on a total of 2507 female and 2216 male wDah flies as well as 3598 females and 3486 males of w1118.

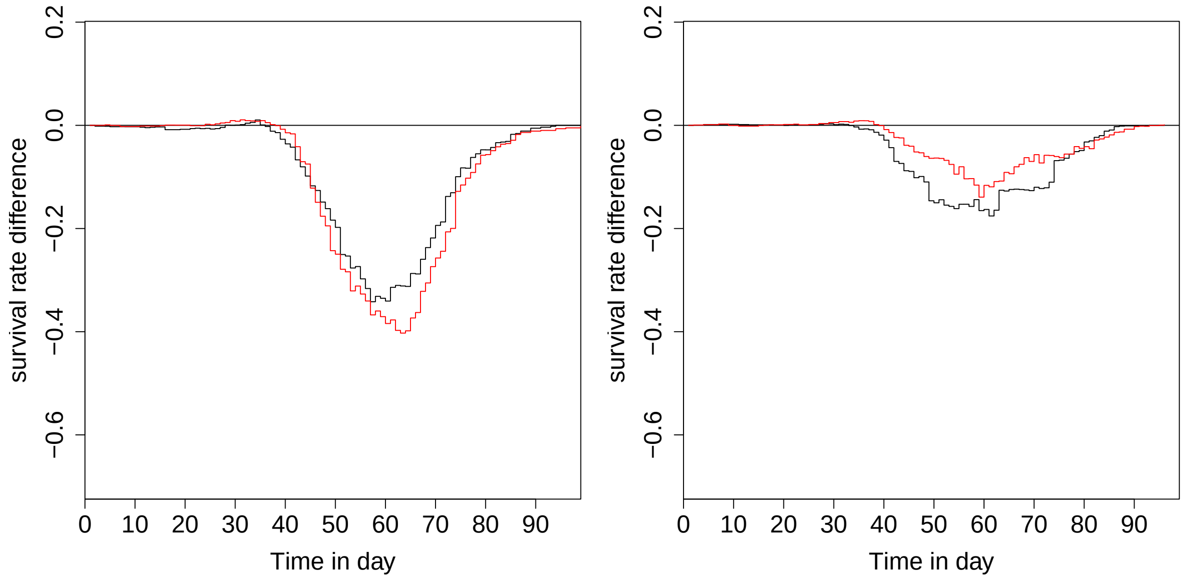

Figure S2: **Gender Difference Profiles of Control and Mutants separately (A)** Female-male difference profiles of wDah controls (black) and mutants (red). **(B)** Female-male difference profiles of w1118 controls (black) and mutants (red). The profiles have been defined as difference plot of the respectively combined female and male cohorts. If the line is above zero this indicates a male survival advantage, where as if it below it indicates a survival disadvantage for male, i.e. a survival advantage for females.

Table S1: SurvCurv IDs used for the gender difference plots (not all are publicly available).

	**female**	**male**
**w1118**	350+351+352+356+357+358+439+441 +443+445+463+465+1108+1109+1621 +1622+1623+1629+1630+1631	347+348+349+353+354+355+438+440 +442+444+466+467+1074+1077+1625 +1626+1627+1633+1634+1635
**wDah**	202+362+363+364+1080+1112+1082 +1113+257+258+259+468+470+519 +520+521+1110+1111	201+359+360+361+1076+1079+254 +255+256+471+472+534+535+536 +1075+1078

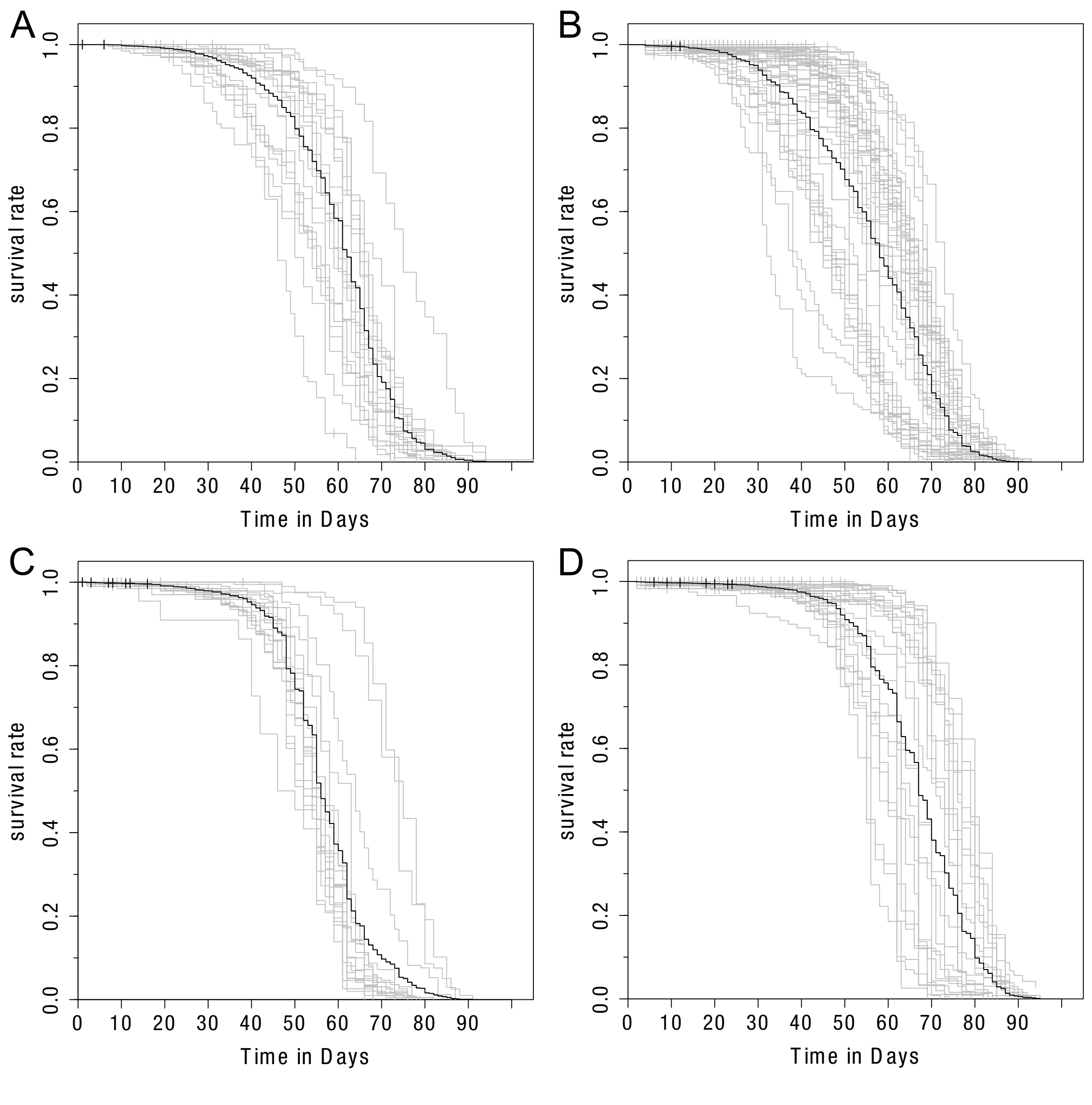

Figure S3: **Variation of female *Drosophila* controls in different strains (A)** Survival curves of 22 female *white* Dahomey (wDah) control cohorts without any genetic constructs. **(B)** Survival curves of 54 female *white* Dahomey (wDah) control cohorts with ostensibly inactive genetic constructs. **(C)** Survival curves of 19 female w1118 control cohorts without any genetic constructs. **(D)** Survival curves of 26 female w1118 control cohorts with ostensibly inactive genetic constructs. Control cohort of all strains where on 1SY food 25 °C. The black line represents the survival curve estimated from the pooled data for each group.

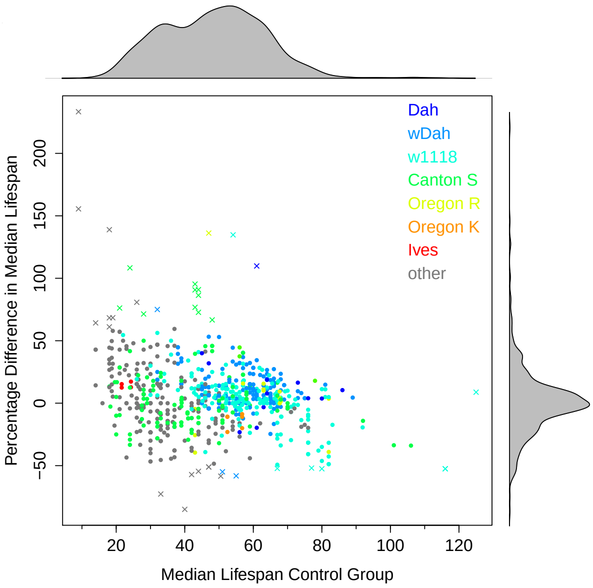

Figure S4: **Correlation analysis of median lifespan and percentage lifespan extension in flies** Correlation of median control lifespans with percentage difference in median lifespan. x indicate outliers excluded for the correlation. Outliers were defined as being more than 3.3 times the MAD estimator away from the mean. Different strains are indicated by different colours and density distribution of the data is shown along the axis.
For pairs from the database: Spearman's correlation coefficient $r_S = -0.085$ [$r_S = -0.11$ including outliers] with wDah $r_S = -0.24$ and w1118 $r_S = -0.44$ respectively.
Using the extended data set Spearman's correlation coefficient of $r_S = -0.11$ [$r_S = -0.15$ including outliers] with wDah $r_S = -0.17$ and w1118 $r_S = -0.56$ respectively.

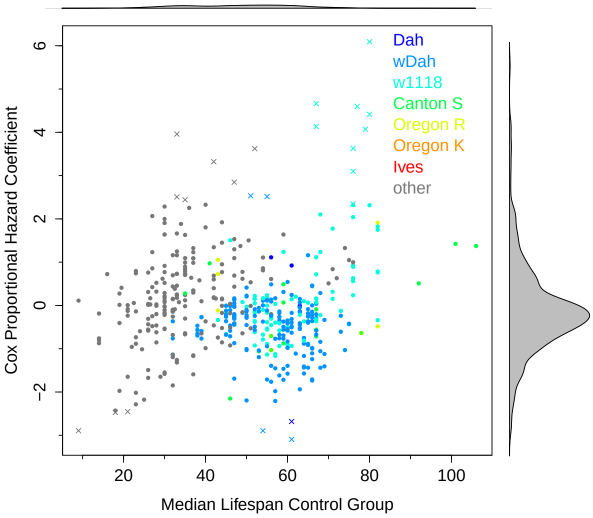

Figure S5: **Correlation analysis of median lifespan and Cox proportional hazards coefficient for control-treatment difference in flies** Correlation of median control lifespans with Cox proportional hazards coefficient for a control-treatment indicator variable. x indicate outliers excluded for the correlation. Outliers were defined as being more than 3.3 times the MAD estimator away from the mean. Different strains are indicated by different colours and density distribution of the data is shown along the axis. As single data point with CoxPH coefficient -21.03 and median lifespan of the control 21 (an outlier) is excluded from plotting.
