# Supplementary material for: Analysing variation in Drosophila aging across independent experimental studies: a meta-analysis of survival data
Source: Aging Cell. 2013 Jul 22;12(5):917–22. doi: 10.1111/acel.12123 (PMC3963443; doi:10.1111/acel.12123)
Supplement: Data S1 [file acel0012-0917-sd2.pdf]

References of articles from which median or mean values were extracted for the analysis lifespan extension and lifespan of the control

Avanesian, A., Khodayari, B., Felgner, J. S. & Jafari, M. 2010 Lamotrigine extends lifespan but compromises health span in *Drosophila melanogaster*. *Biogerontology*, **11**(1), 45–52. (doi:10.1007/s10522-009-9227-1)

Bahadorani, S., Cho, J., Lo, T., Contreras, H., Lawal, H. O., Krantz, D. E., Bradley, T. J. & Walker, D. W. 2010 Neuronal expression of a single-subunit yeast NADH-ubiquinone oxidoreductase (Ndi1) extends *Drosophila* lifespan. *Aging Cell*, **9**(2), 191–202. (doi:10.1111/j.1474-9726.2010.00546.x)

Bauer, J. H., Chang, C., Bae, G., Morris, S. N. S. & Helfand, S. L. 2010 Dominant-negative Dmp53 extends life span through the dTOR pathway in *D. melanogaster*. *Mech Ageing Dev*, **131**(3), 193–201. (doi:10.1016/j.mad.2010.01.007)

Biteau, B., Karpac, J., Supoyo, S., Degennaro, M., Lehmann, R. & Jasper, H. 2010 Lifespan extension by preserving proliferative homeostasis in *Drosophila*. *PLoS Genet*, **6**(10), e1001 159. (doi:10.1371/journal.pgen.1001159)

Chandrashekara, K. T. & Shakarad, M. N. 2011 Aloe vera or resveratrol supplementation in larval diet delays adult aging in the fruit fly, *Drosophila melanogaster*. *J Gerontol A Biol Sci Med Sci*, **66**(9), 965–971. (doi:10.1093/gerona/glr103)

Dubiley, T. A., Rushkevich, Y. E., Koshel, N. M., Voitenko, V. P. & Vaiserman, A. M. 2011 Life span extension in *Drosophila melanogaster* induced by morphine. *Biogerontology*, **12**(3), 179–184. (doi:10.1007/s10522-010-9308-1)

Hao, L.-Y., Giasson, B. I. & Bonini, N. M. 2010 DJ-1 is critical for mitochondrial function and rescues PINK1 loss of function. *Proc Natl Acad Sci U S A*, **107**(21), 9747–9752. (doi:10.1073/pnas.0911175107)

Kim, H.-J., Morrow, G., Westwood, J. T., Michaud, S. & Tanguay, R. M. 2010 Gene expression profiling implicates OXPHOS complexes in lifespan extension of flies over-expressing a small mitochondrial chaperone, Hsp22. *Exp Gerontol*, **45**(7-8), 611–620. (doi:10.1016/j.exger.2009.12.012)

Li, S., Chen, K., Li, X., Zhang, X. & Liu, S. V. 2010 A new cultivation system for studying chemical effects on the lifespan of the fruit fly. *Exp Gerontol*, **45**(2), 158–162. (doi:10.1016/j.exger.2009.11.004)

Mair, W., McLeod, C. J., Wang, L. & Jones, D. L. 2010 Dietary restriction enhances germline stem cell maintenance. *Aging Cell*, **9**(5), 916–918. (doi:10.1111/j.1474-9726.2010.00602.x)

Moskalev, A. A., Plyusnina, E. N. & Shaposhnikov, M. V. 2011 Radiation hormesis and radioadaptive response in *Drosophila melanogaster* flies with different genetic backgrounds: the role of cellular stress-resistance mechanisms. *Biogerontology*, **12**(3), 253–263. (doi:10.1007/s10522-011-9320-0)

Plyusnina, E. N., Shaposhnikov, M. V. & Moskalev, A. A. 2011 Increase of *Drosophila melanogaster* lifespan due to D-GADD45 overexpression in the nervous system. *Biogerontology*, **12**(3), 211–226. (doi:10.1007/s10522-010-9311-6)

Robinson, R. A. S., Kellie, J. F., Kaufman, T. C. & Clemmer, D. E. 2010 Insights into aging through measurements of the *Drosophila* proteome as a function of temperature. *Mech Ageing Dev*, **131**(9), 584–590. (doi:10.1016/j.mad.2010.08.004)

Sanz, A., Soikkeli, M., Portero-Otin, M., Wilson, A., Kemppainen, E., McIlroy, G., Ellila, S., Kemppainen, K. K., Tuomela, T. *et al.* 2010 Expression of the yeast NADH dehydrogenase Ndi1 in *Drosophila* confers increased lifespan independently of dietary restriction. *Proc Natl Acad Sci U S A*, **107**(20), 9105–9110. (doi:10.1073/pnas.0911539107)

Sarkar, M., Iliadi, K. G., Leventis, P. A., Schachter, H. & Boulianne, G. L. 2010 Neuronal expression of Mgat1 rescues the shortened life span of *Drosophila* Ngat11 null mutants and increases life span. *Proc Natl Acad Sci U S A*, **107**(21), 9677–9682. (doi:10.1073/pnas.1004431107)

Schriner, S. E., Katoozi, N. S., Pham, K. Q., Gazarian, M., Zarban, A. & Jafari, M. 2011 Extension of *Drosophila* lifespan by Rosa damascena associated with an increased sensitivity to heat. *Biogerontology*. (doi:10.1007/s10522-011-9357-0)

Sun, X., Seeberger, J., Alberico, T., Wang, C., Wheeler, C. T., Schauss, A. G. & Zou, S. 2010 Açai palm fruit (*Euterpe oleracea Mart.*) pulp improves survival of flies on a high fat diet. *Exp Gerontol*, **45**(3), 243–251. (doi:10.1016/j.exger.2010.01.008)

Vigne, P. & Frelin, C. 2010 Food presentation modifies longevity and the beneficial action of dietary restriction in *Drosophila*. *Exp Gerontol*, **45**(2), 113–118. (doi:10.1016/j.exger.2009.10.016)
